# High-Throughput Sequencing and *De Novo* Assembly of *Brassica oleracea* var. *Capitata* L. for Transcriptome Analysis

**DOI:** 10.1371/journal.pone.0092087

**Published:** 2014-03-28

**Authors:** Hyun A. Kim, Chan Ju Lim, Sangmi Kim, Jun Kyoung Choe, Sung-Hwan Jo, Namkwon Baek, Suk-Yoon Kwon

**Affiliations:** 1 Green Bio Research Center, Korea Research Institute of Bioscience and Biotechnology (KRIBB), Yuseong-gu, Daejeon, Republic of Korea; 2 SEEDERS, Daeduk Industry-Academic Cooperation Building, Gwanpyeong-dong Yuseong-gu, Daejeon, Republic of Korea; 3 Samsung Seed Co., Ltd., Madoo2-ri, Seotan, Pyongtaek, Kyeonggi, Republic of Korea; 4 Biosystems and Bioengineering Program, University of Science and Technology, Daejeon, Republic of Korea; Seoul National University, Republic of Korea

## Abstract

**Background:**

The cabbage, *Brassica oleracea* var. *capitata* L., has a distinguishable phenotype within the genus *Brassica*. Despite the economic and genetic importance of cabbage, there is little genomic data for cabbage, and most studies of *Brassica* are focused on other species or other *B. oleracea* subspecies. The lack of genomic data for cabbage, a non-model organism, hinders research on its molecular biology. Hence, the construction of reliable transcriptomic data based on high-throughput sequencing technologies is needed to enhance our understanding of cabbage and provide genomic information for future work.

**Methodology/Principal Findings:**

We constructed cDNAs from total RNA isolated from the roots, leaves, flowers, seedlings, and calcium-limited seedling tissues of two cabbage genotypes: 102043 and 107140. We sequenced a total of six different samples using the Illumina HiSeq platform, producing 40.5 Gbp of sequence data comprising 401,454,986 short reads. We assembled 205,046 transcripts (≥ 200 bp) using the Velvet and Oases assembler and predicted 53,562 loci from the transcripts. We annotated 35,274 of the loci with 55,916 plant peptides in the Phytozome database. The average length of the annotated loci was 1,419 bp. We confirmed the reliability of the sequencing assembly using reverse-transcriptase PCR to identify tissue-specific gene candidates among the annotated loci.

**Conclusion:**

Our study provides valuable transcriptome sequence data for *B. oleracea* var. *capitata* L., offering a new resource for studying *B. oleracea* and closely related species. Our transcriptomic sequences will enhance the quality of gene annotation and functional analysis of the cabbage genome and serve as a material basis for future genomic research on cabbage. The sequencing data from this study can be used to develop molecular markers and to identify the extreme differences among the phenotypes of different species in the genus *Brassica*.

## Introduction

Crops of the genus *Brassica* (tribe Brassiceae) are commonly used in many foods. The model organism *Arabidopsis thaliana* is a member of the Brassicaceae family. *Brassica oleracea*, one of the most important crops in the genus *Brassica*, is a cruciferous vegetable that is native to coastal southern and western Europe. A number of the most widely consumed cruciferous vegetables are cultivars of *B. oleracea*: Chinese broccoli, cabbage, Brussels sprouts, kohlrabi, broccoli, cauliflower, and others. The *botrytis*, *capitata*, *gemmifera*, *gongylodes*, *italica*, and *medullosa* subspecies of *B. oleracea* are known for their extreme morphological differences [1].

*B. oleracea* is a diploid species with a CC-type genome containing nine chromosomes: x  =  9 (2x  =  2n  =  18) [2]. The estimated size of the *B. oleracea* genome ranges from 599 Mb to 868 Mb [3]–[6], which is four to six times the size of the Arabidopsis genome, 135 Mb, reported by the Arabidopsis Genome Initiative (AGI) [7]. Since 2004, whole-genome shotgun sequencing and BAC end sequencing studies of the *B. oleracea* genome were registered by JCVI (J. Craig Venter Institute) [8] and the *B. oleracea* genetic mapping project at NCBI (National Center for Biotechnology Information). Nevertheless, there are only 106 nucleotide sequences, 24 ESTs, and 57 protein sequences available for *B. oleracea* at NCBI as of August 2013. Cabbage (*B. oleracea* var. *capitata* L.), a type of leafy green vegetable, is one of the six cultivated subspecies of *B. oleracea* and is cultivated in large areas throughout the world. It is a herbaceous, biennial, dicotyledonous flowering plant distinguished by a short stem upon which a mass of leaves is crowded. Approximately 58 tons of cabbage and other *Brassica* species are produced worldwide annually, ranking *Brassica* among the top 20 commodities in the world [9]. Despite the economic importance and the distinctive genetic features of cabbage, genome-scale or transcriptome-scale research on cabbage is sparse.

RNA-Seq is a powerful, recently developed, high-throughput sequencing method that uses deep sequencing to produce millions of short sequence reads, enabling gene expression profiling that reveals many novel transcribed regions, splice isoforms, single nucleotide polymorphisms (SNPs), and precise locations of transcription boundaries. Expressed sequence tags (ESTs) are partial sequences derived from complementary DNA (cDNA). ESTs represent gene expression in the samples and several ESTs could be generated from a single gene [10]. Full-length cDNAs, representing the entire transcription unit, are more useful than partial sequences for transcriptome analysis and genome annotation [11]–[14]. Full-length cDNAs can be constructed and selected based on the 5′-cap, a distinctive feature of mRNA structure [15]–[18]. Moreover, the genes predicted from *de novo* assemblies must be validated to ensure the efficacy of the assemblies. Because reverse-transcriptase PCR (RT-PCR) facilitates the detection and quantification of target mRNA transcripts, we used RT-PCR to identify tissue-specific gene candidates in order to validate the reliability of our cabbage transcriptome assembly. Using RT-PCR to identify the tissue-specific genes predicted by *de novo* assembly and analysis of deep-sequencing data could be a means to experimentally validate the existence of the assembled genes. Tissue-specific genes are preferentially expressed and function in specific tissues or cell types, providing not only experimental validation of genes assembled *de novo*, but also spatial or time-course expression patterns, showing where and when specific genes are working. Thus, the information allows us to infer relationships between tissues and genes, temporal or growth stage-specific gene expression, and novel gene functions [39].

In this study, 401,454,986 short reads were produced using the Illumina HiSeq platform. The reads were assembled into 205,046 transcripts and 53,562 loci, 35,274 of which had homology with peptide sequences in the Phytozome database, and 11,438 of which were full length. Also, tissue-specific gene candidates were predicted and sorted. The sequences of the loci and the annotation data from this study will be useful resources for the ongoing cabbage whole-genome sequencing project and the characterization of gene expression patterns and traits of cabbage and closely related species.

## Materials and Methods

### Plant materials and RNA extraction

We generated sequence libraries for two cabbage cultivars provided by Samsung Seed Co. From cultivar 107140 (accession number from Samsung Seed Co.), we collected a 9-day-old seedling grown *in vitro* under normal conditions, a 14-day-old seedling grown *in vitro* under normal conditions for 9 days and under calcium-deficient conditions for 5 days, roots from seedlings grown *in vitro*, and leaves from plants grown in a greenhouse. From cultivar 102043 (accession number from Samsung Seed Co.), we collected flowers from plants grown in a greenhouse and a 9-day-old seedling grown *in vitro* under normal conditions. Total RNA was isolated from each sample using the QIAGEN RNeasy Mini Kit according to the manufacturer's instructions. The RNeasy MinElute Cleanup Kit (Qiagen) was used to remove residual DNA from each sample. The quality and quantity of the RNA were measured using a Nanodrop ND-1000 spectrophotometer. Purified RNA was used to synthesize cDNA.

### mRNA sequencing, *de novo* assembly, and annotation

**Figure 1:**
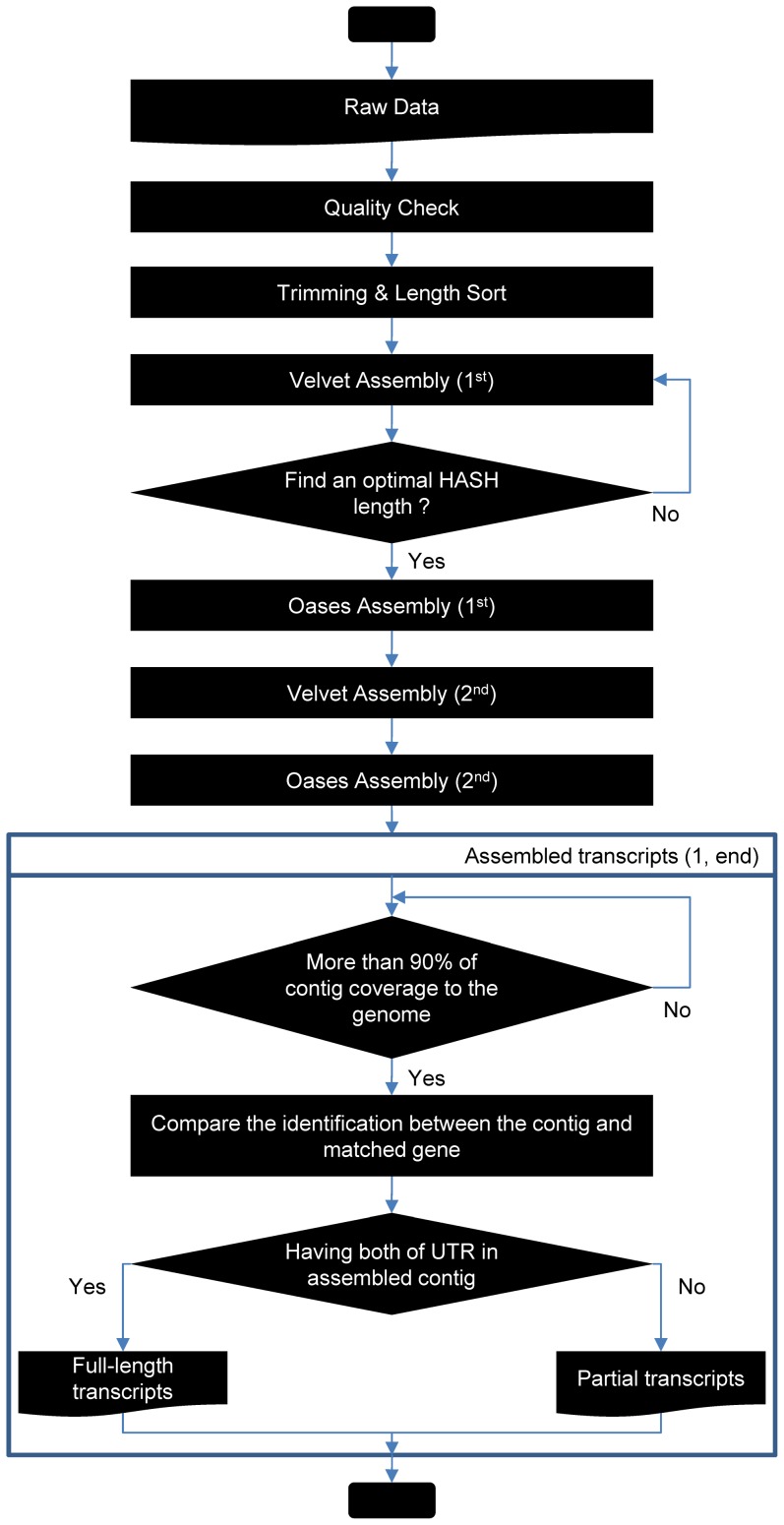
Workflow of the transcriptome assembly and the analysis of high-throughput sequencing data. The analysis of the transcriptome assembly and the full-length transcripts were processed as a workflow. The quality analysis of the sequence data, the data trimming, and the read length sorting were performed by the Solexa QA, Dynamic Trim, and Length sort programs, respectively. The optimal hash length for the assembly was selected by applying several hash lengths according to an in-house pipeline. The assembled transcripts with more than 90% coverage of the Arabidopsis genome were analyzed to identify full-length transcripts. The transcripts with both a 5′UTR and a 3′UTR were defined as full-length transcripts (fl-transcripts).

We used 5 μg total RNA from each sample to create normalized cDNAs. The cDNAs were amplified according to the Illumina RNA-Seq protocol and sequenced using the Illumina HiSeq 2000 system, producing 40.5 Gbp of 101-bp paired-end reads. We extracted the sequence data for the base pairs with quality scores of Q ≥ 20 using SolexaQA [19]. We used all the sequence reads from the different tissue samples to optimize *de novo* assembly using two software tools based on the de Bruijn graph algorithm. We used Velvet (v1.2.07) [20] to assess *k*-mer sizes and assemble contigs. We joined the contigs into transcript isoforms using Oases (v0.2.08), which was specially developed for the *de novo* assembly of transcripts using short reads [21]. We considered several hash lengths to select the best *de novo* assembly. A schematic design of the process is shown in Figure 1. We validated the transcripts assembled from the total reads merged from each mRNA sample by direct comparison with gene sequences in the Phytozome database (http://www.phytozome.net/) using BLASTX (e-value ≤ 1e^−05^). We retrieved the protein sequences with the highest sequence similarity for further analysis.

### Functional enrichment analysis

For Gene Ontology [24] term analysis, GO database (http://www.geneontology.org/) was downloaded and cabbage loci were annotated to the GO database using BLASTP (e-value = 1e^−06^). Map2Slim.pl script was applied to retrieve the GO term annotation result and the number of cabbage loci assigned with GO term was counted using in-house scripts of SEEDERS Co. We carried out functional enrichment analysis using DAVID, a web-accessible program providing a comprehensive set of functional annotation tools for inferring biological meaning from large lists of genes [22], [23]. We analyzed the gene lists annotated with the TAIR IDs of the transcripts using the default criteria (counts ≥ 2 and EASE score ≤ 0.1) Kyoto Encyclopedia of Genes and Genomes (KEGG) pathway [25].

### Short-reads counting and tissue-specific reverse-transcriptase PCR

We sequenced the mRNA libraries generated from each sample of two cultivars using Illumina HiSeq2000 (101 bp paired-end). The reads for each sequenced tag were mapped to the assembled loci using Bowtie (mismatch ≤ 2 bp), and the number of clean mapped reads for each locus was counted. We selected tissue-specific genes based on the read counts from the leaf and root samples of cultivar 107140 and the flower sample of cultivar 102043. The criteria for selecting tissue-specific gene candidates were that the number of mapped reads should be more than 100 in the target tissue and less than 10 in the other tissues. We identified 30 tissue-specific genes, 10 genes from each sample, used them for RT-PCR. The tissue-specific genes and corresponding primers are shown in Tables S7 and S8. *B. oleracea* actin (AF044573) was used as a control, and the primer sequences were 5TGGTTGGGATGAACCAGAAG-3 and 5- CCAGAGTCCAGCACAATACC-3. Except for those used for Locus_39612, Locus_13581 and Locus_29088, the RT-PCR conditions were: denaturation at 95°C for 5 min, followed by 26 cycles of denaturation at 95°C for 30 s and annealing at 58°C for 30 s. For Locus_39612 and Locus_13581, we used an annealing temperature of 55°C, and for Locus_29088, we performed 33 cycles with an annealing temperature of 58°C. The RT-PCR products were electrophoresed on 1.5% agarose gel containing ethidium bromide.

## Results and Discussion

### Cabbage transcriptome sequencing and *de novo* assembly

Cultivar 107140 had a thicker wax layer on the leaves and a smaller head size than cultivar 102043. In future studies, the characteristics of each cultivar will be treated in relation to the transcriptomic data produced by this study. From the six different tissues, 40.5 Gbp (401,454,986 raw reads) were generated (Table 1). Because removing low-quality bases at the ends of reads and assembling only high-quality reads improves the assembly significantly [26], we checked the quality of the sequence data (Q ≥ 20) using SolexaQA, and we trimmed and sorted the reads by length using the DynamicTrim and LengthSort programs [19]. Similar to trimming the low-quality bases at the end of reads, merging the contigs generated by multiple assemblies can also enhance the assembly results [27], [28]. We applied two software tools, Velvet and Oases, based on de Bruijn graphs. The assembly results of the de Bruijn graph-based assemblers depend strongly on two parameters: the *k*-mer length and the value of the coverage cutoff. Because different *k*-mer lengths and coverage cutoffs generate different assembly results [26], [29], [30], we assessed the performance of different *k*-mer lengths using raw reads data before performing the *de novo* assembly. To select the optimal hash length, we performed *de novo* assembly using *k*-mer lengths from 51 to 63 (Table 2).Considering N50, average contig length, max length, the number of contigs, and total length, we concluded that *k* -mer  =  57 and *k*-mer  =  59 represented high connectivity of contigs and stable gene-sequence, respectively and finally selected *k*-mer 57, and 59 for our assembly. We combined the transcripts generated by Velvet and Oases using *k*-mer  =  57 and *k*-mer  =  59 and assembled them again using Velvet followed by Oases to construct extended transcripts. First, 86,617 and 84,564 transcripts were produced by Velvet and Oases with *k*-mer  =  57 and *k*-mer  =  59, respectively. From those transcripts, 205,046 extended transcripts (≥ 200 bp) were built using *k*-mer  =  57 and *k*-mer  =  59 (Table 3). The average length of the extended transcripts was 1,434 bp, and the lengths of the extended transcripts ranged from 200 bp to 16,439 bp (Table 1). Finally we predicted 53,562 loci from the extended transcripts. We annotated 35,274 of the predicted loci with 26,970 plant peptide sequences from the Phytozome database (http://phytozome.net/). The average length of the annotated loci was 1,419 bp (Table 3).

**Table 1:** Summary of short-read data from cabbage produced using Illumina HiSeq.

	Cabbage
**Number of tissues**	6
**Number of raw reads**	401,454,986
**Number of raw bases**	40,546,953,586
**Number of reads assembled**	282,823,640
**Number of bases assembled**	23,662,266,690
**Number of assembled transcripts (** ***k-mer*** ** = 57)**	205,046
**Number of assembled loci**	53,562
**Mean transcript length (bp)**	1,434
**Range of transcripts lengths**	200 ∼ 16,439

**Table 2:** Summary statistics of the assemblies of the cabbage sequence data showing the performances of the multiple-k *de novo* assemblies.

K-mer^1^	Contig ≥ 200	N50^2^	Average length (bp)^3^	Max length^4^	Total Length (Mb)^5^
51	94,085	695	553	15,460	52
53	91,543	716	557	13,186	51
55	88,889	742	574	14,732	51
57	86,617	764	577	14,732	50
59	84,564	776	579	14,490	49
61	84,425	790	580	13,567	49
63	82,079	807	597	14,228	49
57 + 59 + OASES	205,046	1,915	1,434	16,439	294

1 *k-mer*: Required length of identical overlap match between two reads by Velvet.

2 N50: Contig length-weighted median.

3 Average length: length of a contig  =  the number of contigs/total length.

4 Max length: Length of the longest contig.

5 Total length: Summed length of all contigs.

**Table 3:** Results of the cabbage *de novo* assembly using Velvet and Oases.

Source	Description	Number
Velvet	Contigs (*k-mer* = 57, 59)	171,181
	Average contig length	580
OASES	Extended contigs (*k-mer* = 57, 59)	205,046
	Loci ≥ 200 bp	53,562
	Loci (annotation)	35,274
	Number of annotated genes	26,970
	Average annotated transcripts length	1,419

### Functional annotation and characterization of the cabbage transcripts

**Figure 2:**
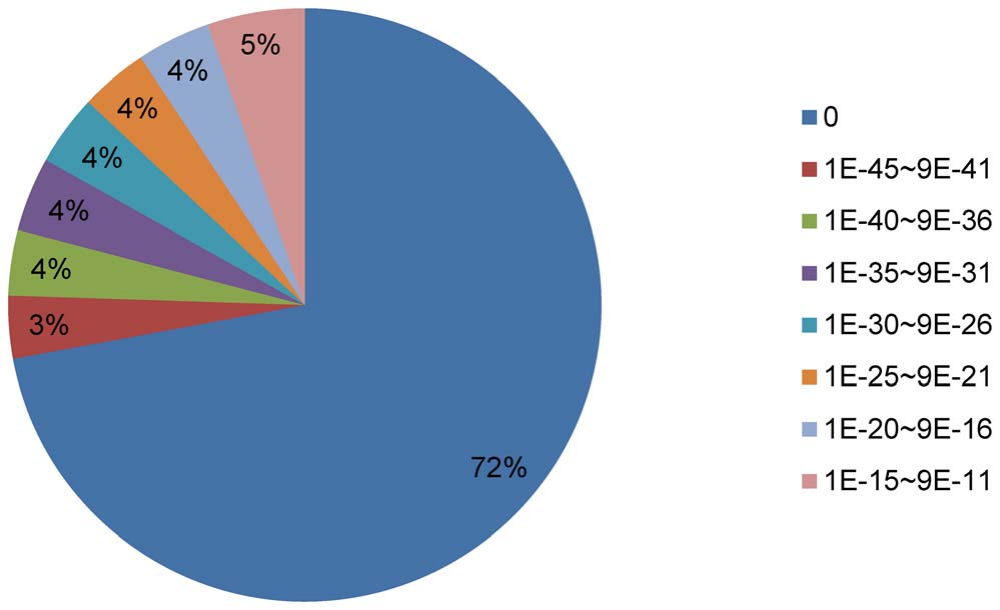
E-values of the cabbage loci annotation. We annotated 35,274 of 53,562 cabbage loci (65.9%) with 26,971 plant peptide sequences from the Phytozome database. The e-values of 25,472 of the cabbage loci were equal to zero, accounting for more than 72% of the annotated loci.

To identify the putative functions of the transcripts, we used BLASTX to compare the 53,562 predicted loci to the 1,232,565 sequences in the Phytozome database, which contains 31 sequenced plant genomes annotated with PFAM, KOG, KEGG, and PANTHER assignments and linked to annotations in RefSeq, UniProt, TAIR, and JGI. We annotated 35,274 of the predicted loci (65.8%) with 26,970 plant peptide sequences from the Phytozome database (http://phytozome.net). The average length of the annotated loci was 1,419 bp (Table 3). Many of the loci were homologous to uncharacterized proteins or housekeeping genes (Table S1). Seventy-two per cent (25,472) of the annotated cabbage loci had an e-value of zero, which is significantly more than in previous *de novo* sequencing reports [31], [32]. Higher sequence homology between assembled loci and annotated reference genes provides more reliable putative functions for the loci and reduces the labor required to identify and authenticate putative gene functions. The high number of annotated loci with an e-value of zero in our dataset reflects the validity and reliability of our *de novo* assembly (Figure 2).

**Figure 3:**
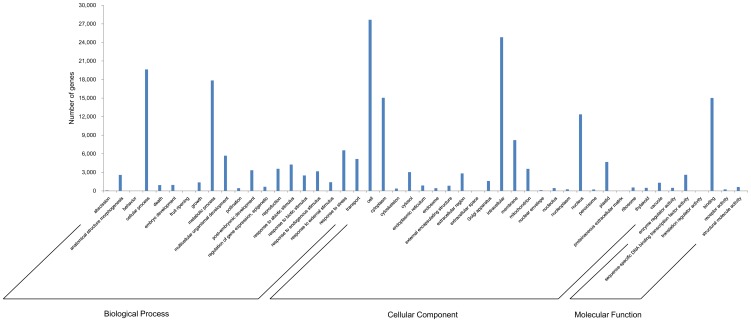
Histogram of the GO classification. The cabbage loci were annotated in three ontology categories: ‘Biological Processes’, ‘Cellular Component’, and ‘Molecular Function’.

We assigned Gene Ontology (GO) [24] terms to the cabbage loci. The GO database is a major bioinformatics initiative to develop and use ontologies to support biologically meaningful annotation of genes and gene products in a wide variety of organisms. We assigned GO terms to the 33,022 annotated loci. The GO terms represented 46 functional categories. Twenty ‘Biological Process’ categories were assigned among 30,325 cabbage loci; Twenty-three ‘Cellular Component’ categories were assigned among 31,031 cabbage loci; and six ‘Molecular Function’ categories were assigned among 29,718 cabbage loci (Figure 3). Because many of the transcripts were assigned more than one GO term, the total number of assigned GO terms was larger than the total number of annotated loci. ‘Metabolic Process’ (58.8%) and ‘Cellular Process’ (64.7%) were the most common terms in the ‘Biological Process’ category; ‘Cell’ (89.1%) and ‘Intracellulart’ (80.0%) were the most common terms in the ‘Cellular Component’ category; and ‘Binding’ (50.5%) was the most common term in the ‘Molecular Function’ category (Table S2). The large proportions of certain GO terms among the annotated loci may reflect high levels of conservation in genes performing similar functions in different species, making those genes easier to annotate in the database.

**Figure 4:**
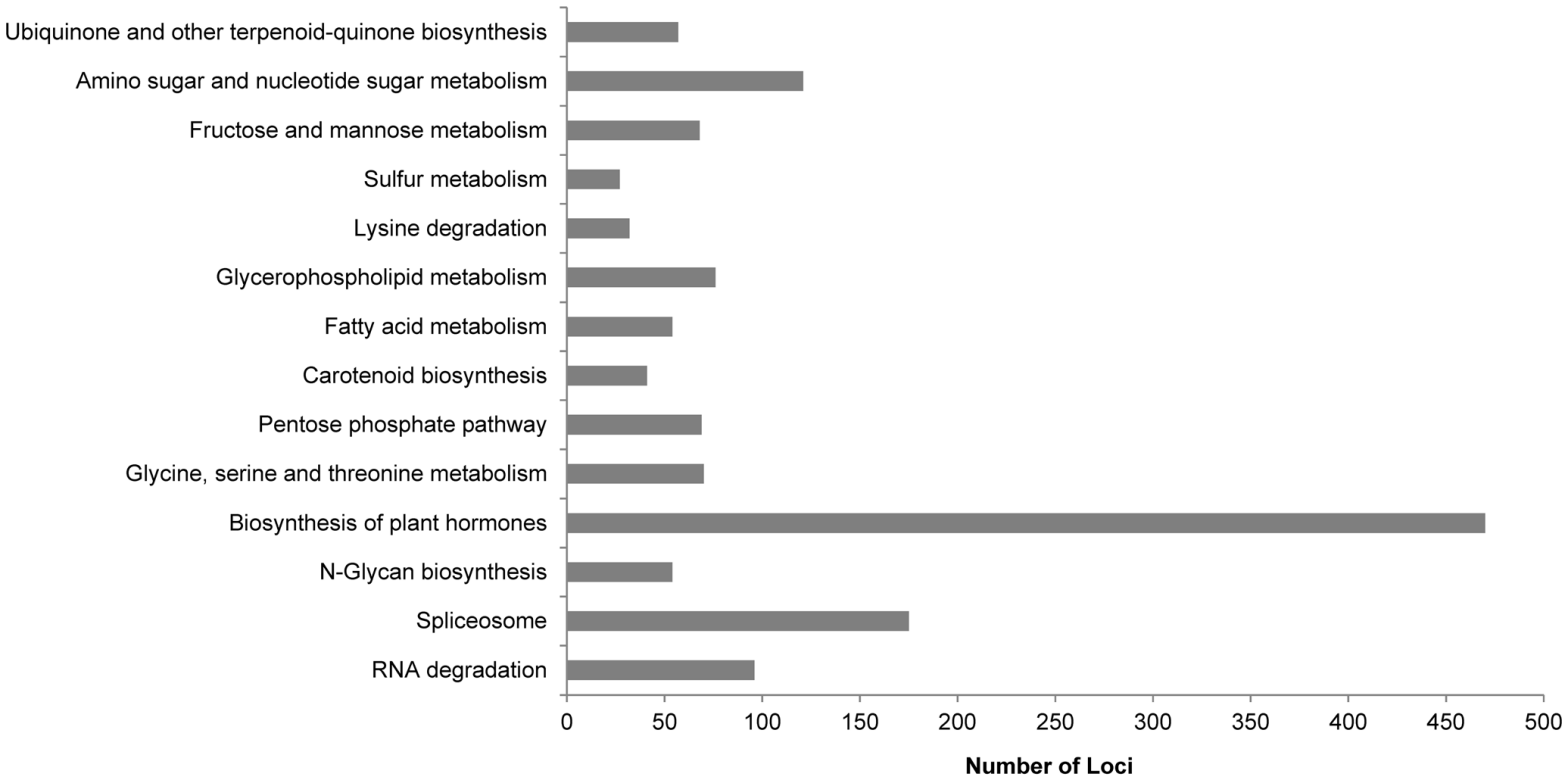
KEGG annotation of the cabbage assembly. KEGG annotation was performed using 18,761 TAIR IDs; 733 of the TAIR IDs covered 14 KEGG pathways. The 1,410 cabbage loci annotated by those TAIR IDs were sorted to the corresponding KEGG pathways.

To find genes involved in important pathways, we assigned 18,761 TAIR IDs to the annotated cabbage loci using DAVID [22], [23] and then used the TAIR IDs to annotate the loci with Kyoto Encyclopedia of Genes and Genomes (KEGG) pathways [25]. We sorted 733 which were assigned to 1,410 cabbage loci, to 14 KEGG pathways (Figure 4). The largest number of cabbage loci (470 loci) were annotated with 87 Enzyme Codes (ECs) linking them to the ‘Biosynthesis of Plant Hormones’ KEGG pathway. In total, 1,410 total loci were annotated with 452 ECs, of which 211 were unique ECs (Table S3).

To screen Single Nucleotide Polymorphisms (SNPs) between the two different cabbage cultivar, 107140 and 102043, cabbage loci which were predicted to be Differentially Expressed Genes (DEGs) by the number of read-count were selected. Of the loci, when the base differs from each other, we only considered it as SNPs between two cabbage cultivars. Also SNPs between high quality base pairs were primarily compared and if there was low quality base pair, it was marked in lowercase (Table S4).

### Gene coverage and length distribution of the *de novo* assembly

**Figure 5:**
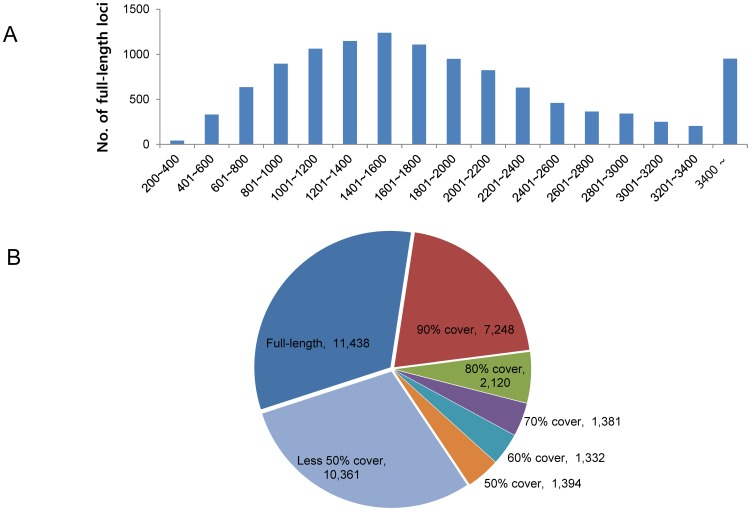
Length distribution and reference gene coverage rate of the full-length cabbage loci. Of the 35,274 loci annotated with genes from the Phytozome database using BLAST, 11,438 loci were predicted to be full-length loci. (A) The minimum length was 226 bp, and the maximum length was 16,439 bp. The largest number of full-length loci was in the range of 1,201 ∼ 1,500 bp. (B) Pie chart of the 35,274 loci classified by percentage of coverage on the reference gene.

We refer to gene coverage as the number of bases within an assembled locus that can be matched to a single reference gene. The gene coverage information is useful for selecting genes of interest for functional experiments, because loci with low gene coverage may not function as expected based on the information about the reference gene. That does not mean that partial transcripts are dispensable, however, because partial transcripts can be applied to investigate alternative splicing, RNA editing, new transcript isoforms, and for other purposes. We regarded loci covering 90% of a reference gene sequence as full-length loci. We sorted 35,274 annotated loci by gene coverage in the Phytozome database and found that 24,913 of the annotated loci (70.6%) covered ≥ 50% of the reference genes in the database (Figure 5A). In other words, about half (52.9%) of the 35,274 loci covered more than 90% of the annotated genes. Among the 35,274 annotated cabbage loci, 11,438 (32.4%) were annotated with 18,799 sequences from the Phytozome database and were full-length loci, and 23,836 (67.6%) were annotated with 37,117 sequences from the Phytozome database and were partial loci (Table 4). The average length of the annotated loci was 1,419 bp, which was similar to results previously reported for tomato (1,418 bp; [33]) and soybean (1,539 bp; [34]). The average number of assembled loci per assembled transcript, 26.1, was lower than that reported by other studies (e.g., Xiang Tao et al. reported an average of 40.4) [35]. The reason for the higher number in our study may be that we only used loci longer than 200 bp, and 193,984 of our loci were shorter than 200 bp, whereas previous studies used transcripts as short as 100 bp in length. The lengths of the 11,438 full-length loci in our study ranged from 226 to 16,439 bp, and the largest number of full-length loci had lengths in the range 1,201 ∼ 1,400 bp (Figure 5B). With the e-value distribution of the 35,274 annotated loci shown in Figure 2, the gene coverage percentage of the full-length loci supports the reliability of our *de novo* assembly.

**Table 4:** Full-length ratio of the assembled cabbage transcripts.

	VELVET			Phytozome
No. of Loci	53,562			1,232,565
	Homology	35,274 (65.9%)		55,916
		Full-length	11,438 (32.4%)	18,799
		Partial-length	23,836 (67.6%)	37,117
	Others	18,288		–

### Expression of tissue-specific locus candidates

Tissue-specific genes are preferentially expressed in one or more specific tissues or cell types. Spatial or time-course expression of genes provides information about where and when the genes are working. Measuring tissue-specific expression allows us to infer tissue-gene relationships and temporal or growth stage-specific gene expression, potentially revealing novel gene functions [36]. Because RT-PCR facilitates the detection and quantification of target mRNA transcripts, we performed RT-PCR with tissue-specific genes to validate the reliability of our cabbage transcriptome assembly.

We classified the cabbage loci assembled from the leaf and root samples of cultivar 107140 and the flower sample of cultivar 102043 by the number of reads annotated with GO terms specific to each tissue type. The tissue-specific loci and GO terms are listed in Tables S5 and S6, respectively. Specifically, the GO categories of the flower-specific candidates included ‘Reproductive Developmental Process’, ‘Reproductive Process’ and ‘Post-embryonic Development’. We collected 10 tissue-specific candidate loci from each of the three tissues (Table S7), and we designed primer sets for the candidates (Table S8). The RT-PCR results identified several tissue-specific candidate loci that were dominantly expressed in each tissue type, respectively.

We identified six genes that were preferentially expressed in the flower sample: calcium-dependent protein kinase 25 (AT2G35890.1; locus_52607, 1,255 bp), previously shown to be expressed in flowers, plant sperm cells, pollen cells, and pollen tube cells [37], [38]; stigma-specific Stig1 family protein (AT1G53130.1; locus_34045, 799 bp), previously shown to encode a cysteine-rich protein expressed in the stigmatic secretory zone [39]; uncharacterized protein family (UPF0497) (AT3G14380.1; locus_52340, 928 bp), previously shown to be expressed in abscising flower tissues [40]; Cytochrome P450, family 71, subfamily B, polypeptide 31 (AT3G53300.1; locus_52302, 1,823 bp), previously shown to be expressed in the carpel, pollen, sepal, and stamen [41]; K-box region and MADS-box transcription factor family protein (AT1G69120.1; locus_52048, 1,050 bp), previously shown to be expressed in young flower primordial [42]; and MYB domain protein 57 (AT3G01530.1; locus_48530, 1,117 bp), previously shown to be expressed in young flower buds [43].

We identified three genes that were preferentially expressed in the leaf sample: pyridoxal-5’-phosphate-dependent enzyme family protein (AT5G28237.1; locus_49796, 1642 bp), equilibrative nucleoside transporter 3 (AT4G05120.1; locus_13581, 2160 bp), and 2-oxoglutarate (2OG) and Fe (II)-dependent oxygenase superfamily protein (AT4G25300.1; locus_23443, 1285 bp). All three of the leaf-specific genes that we identified were previously shown to be preferentially expressed in the guard cells of leaves [44].

We identified eight genes that were preferentially expressed in the root sample. Nitrate transporter 2∶1 (AT1G08090.1; locus_6549, 1,893 bp) was previously shown to be expressed in the root tissues of Arabidopsis, soybean, and *Nicotiana plumbaginifolia* [45]–[49]. Arabidopsis thaliana low-K^+^-tolerant 1 (AT4G32650.1; locus_9996, 1,468 bp), proline-rich protein 3 (AT3G62680.1; locus_3682, 1,238 bp), and alpha/beta-Hydrolase superfamily protein (AT1G30370.1; locus_44970, 2,005 bp), were previously shown to be expressed in root hairs, root endodermis [50], and roots [41], [51], respectively. Cation/H+ exchanger 17 (AT4G23700.1; locus_3133, 2,772 bp) was previously detected in matured roots using the *E. coli* GUS gene under the control of the 2-kb promoter sequence of AT4G23700.1 [52]. Mildew resistance locus O 15 (AT2G44110.2; locus_29088, 755 bp) was previously shown to be preferentially expressed in the early elongation zone of root tips [53]. Plant U-box 23 (AT2G35930.1; locus_26122, 1,584 bp) was previously shown to be overexpressed in Arabidopsis plants that have longer roots than the wild type, suggesting the possibility that plant U-box 23 is involved in tissue growth during root development [54]. High affinity nitrate transporter 2.6 (AT3G45060.1; locus_8411) was previously shown to be preferentially express in roots [55]. The results of the functional and expression analyses of the tissue-specific candidate loci support the hypothesis that the tissue-specific cabbage loci have the same or similar functions and expression patterns as the previously described reference genes.

We checked the annotations of the *Brassica rapa* transcripts in the EnsemblPlants database (http://plants.ensembl.org/Brassica_rapa/Transcript) for TAIR IDs and tissue-specific expression patterns that matched those of our tissue-specific cabbage loci.

We found six *B. rapa* transcripts in the database with TAIR IDs matching those of our cabbage flower-specific candidate loci and reported to be expressed in the flower tissue. Bra017283.1 (1,548 bp), Bra018871.1 (504 bp), Bra027343.1 (519 bp), Bra006988.1 (1,503 bp), Bra038326.1 (771 bp), and Bra0014005.1 (627 bp) were annotated to AT2G35890.1 (1,563bp), AT1G53130.1 (822bp), AT3G14380.1 (772bp), AT3G53300.1 (1,670bp), AT1G69120.1 (1,228 bp), and AT3G01530.1 (1,507 bp), respectively. Except for locus_52607 (1,255 bp), the lengths of the cabbage loci were longer than those of the *B. rapa* transcripts, and the cabbage loci had e-values equal to zero. The e-values of the *B. rapa* transcripts were 4e^−055^, 1e^−066^, 8e^−200^, and 1e^−123^ for Bra018871.1, Bra027343.1, Bra006988.1, and Bra038326.1, respectively. Although the length of cabbage locus_52607 (1,255 bp) was shorter than that of Bra017283.1 (1,548 bp), the e-value of the cabbage locus was zero and that of the *B. rapa* transcript was 3e^−250^.

We found 12 *B. rapa* transcripts in the database with TAIR IDs matching those of our cabbage leaf-specific candidate loci and reported to be expressed in the leaf tissue. Bra016829.1 (1395 bp; 2e^−222^), Bra016830.1 (1359 bp; 8e^−148^), and Bra037230.1 (1326 bp; 3e^−186^) were annotated to AT5G28237.1 (1578 bp). Bra029554.1 (969 bp; 4e^−089^), Bra02955.1 (1257 bp, 4e^−204^), Bra029556.1 (1257 bp, 3e^−190^), Bra029557.1 (1257 bp, 1e^−221^), and Bra036656.1 (1278 bp, 3e^−208^) were annotated to AT4G05120.1 (1531bp). Bra019177.1 (1065 bp, 1e^−134^), Bra010469.1 (1053 bp, 1e^−142^), Bra010470.1 (804 bp, 9e^−124^), and Bra010472.1 (1077 bp, 1e^−163^) were annotated to AT4G25300.1 (1297bp). In each case, the e-value of the *B. rapa* transcript was higher than that of the corresponding cabbage locus, which was equal to zero.

We found 25 *B. rapa* transcripts in the database with TAIR IDs matching those of our cabbage root-specific candidate loci and reported to be expressed in the root tissue. Bra030713.1 (1,593 bp, 1e^−290^), Bra031610.1 (1,590 bp, 3e^−291^), Bra031611.1 (1,521 bp, 5e^−252^), Bra018655.1 (1,506 bp, 4e^−159^), and Bra018656.1 (1,464 bp, 9e^−219^) were annotated to AT1G08090 (1,900 bp). Bra037049.1 (1,938 bp, 4e^−290^) and Bra011367.1 (1,956 bp, 3e^−292^) were annotated to AT4G32650 (2,194 bp). Bra007693.1 (312 bp, 2e^−042^), Bra003506.1 (1,032 bp, 1e^−066^), Bra014398.1 (936 bp, 3e^−056^), and Bra014399.1 (1,287 bp, 6e^−057^) were annotated to AT3G62680 (1,173 bp). Bra014877.1 (426 bp, 6e^−238^) and Bra032383.1 (528 bp, 9e^−273^) were annotated to AT1G30370 (1,590 bp). Bra037666.1 (2,019 bp, 7e^−204^) was annotated to AT2G44110 (1,496 bp). Bra023044.1 (1,230bp, 9e^−198^), Bra017278.1 (1,239 bp, 2e^−205^), and Bra005309.1 (1,164 bp, 9e^−184^) were annotated to AT2G35930. Bra034142.1 (465 bp, 4e^−075^), Bra037625.1 (1,626 bp, 2e^−296^), Bra037626.1 (1,641 bp, 2e^−256^), Bra038301.1 (1,632 bp, 8e^−254^), and Bra038302.1 (1,617 bp, 1e^−284^) were annotated to AT3G45060. Bra019276, Bra010543, and Bra013724 were annotated to AT4G23700, cation/H+ exchanger 17, with e-values equal to zero. The e-values of the other *B. rapa* transcripts were significantly higher than those of the corresponding cabbage loci, which all had e-values equal to zero. The comparison of the Arabidopsis reference annotations for the *B. rapa* transcripts and the cabbage loci supports the credibility of our *de novo* assembly and annotation.

**Figure 6:**
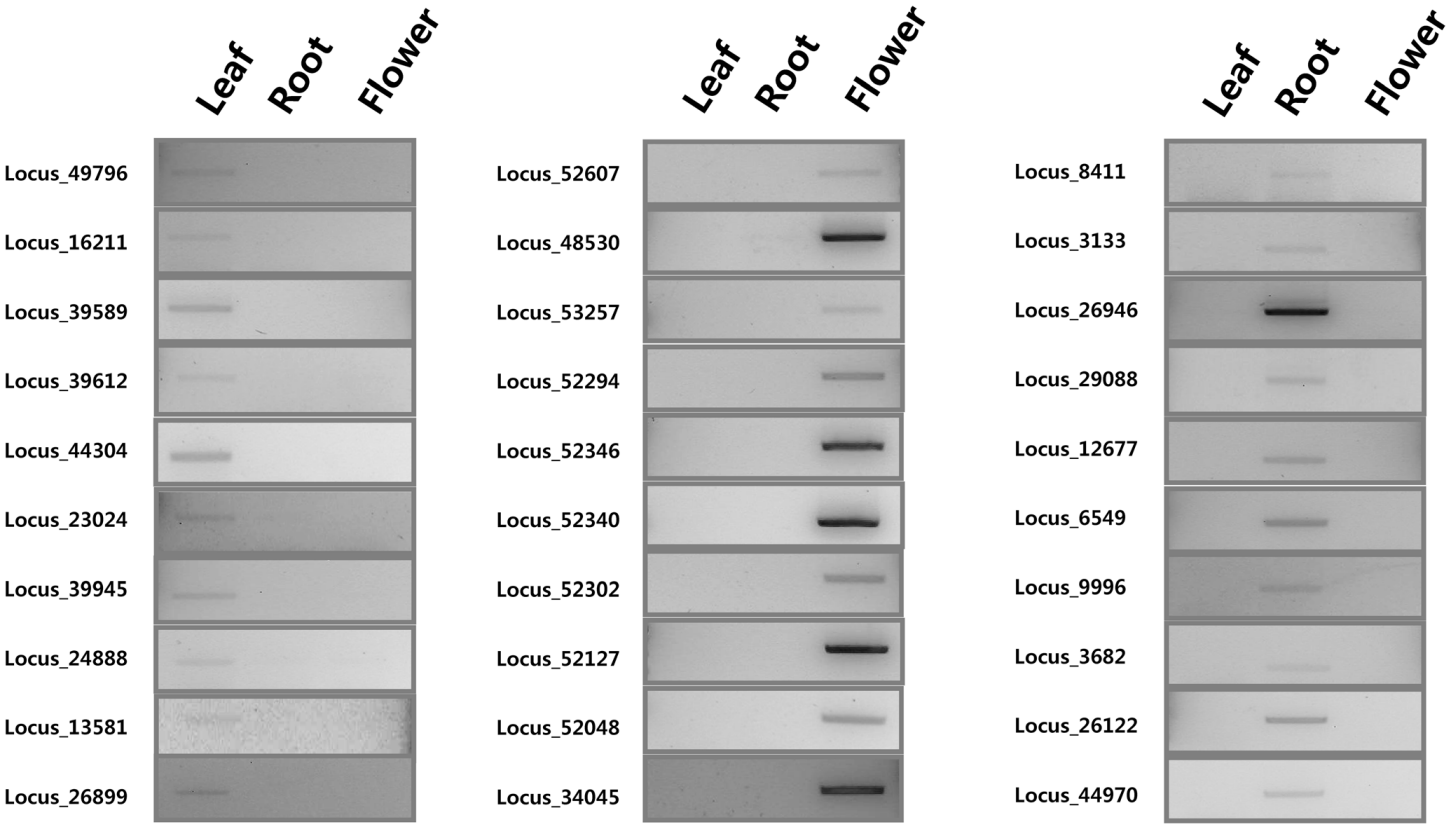
RT-PCR of tissue-specific cabbage genes. RT-PCR was performed with leaf and root samples of cultivar 107140 and the flower sample of cultivar 102043. The RT-PCR results of the leaf-specific (A), flower-specific (B) and root-specific (C) candidate loci are shown.

Each of the 30 tissue-specific cabbage genes selected in our study was preferentially expressed in the target tissue (Figure 6). Experimentally confirmed, tissue-specific genes provide insight into tissue-gene relationships, and they also provide a better understanding of the function and regulation of the genes. Using RT-PCR, we confirmed the tissue-specific gene expression of 30 tissue-specific loci candidates, suggesting that the *de novo* assembly and annotation data from our study can be used in practical experiments in the future.

## Conclusion

High-throughput mRNA sequencing is useful for gene expression profiling in non-model organisms that lack genomic sequence data. Cabbages are a *B. oleracea* subspecies with a basic chromosome number x  =  9 (2x  =  2n  =  18). Although there are some sequencing and functional genomics studies of *B. oleracea* [8], [56]–[60], most genomic or transcriptomic sequencing data from the genus *Brassica* are focused on *B. napus* and *B. rapa*. Even among the sequencing reports on *B.* oleracea, few focus on *B. oleracea* var. *captiata* L., the common cabbage [61]–[63]. Consequently, there is little sequence information on cabbages: as of August 2013, there are only 106 nucleotide sequences, 24 EST sequences, and 57 peptide sequences available from NCBI. We assembled cDNA sequences from six different samples of two cabbage cultivars using the Illumina HiSeq 2000 platform. We assembled 40.5 Gbp sequences comprising 401,454,986 short reads into 171,181 contigs, using Velvet, and 205,046 transcripts, using the Oases assembler. We combined the 205,046 transcripts (≥ 200 bp) into 53,562 loci (Figure S1). We annotated 35,274 of the loci with genes in the Phytozome database, and 11,438 (32.4%) of the transcripts were full-length loci. We assigned the 33,022 annotated cabbage loci to 49 functional groups according to GO classification: 20 biological processes, 23 cellular components, and 6 molecular functions. The ‘Biological Process’, ‘Cellular Component’, and ‘Molecular Function’ GO categories corresponded to 30,235 cabbage loci, 31,031 cabbage loci, and 31,032 cabbage loci, respectively. We performed RT-PCR with 30 cabbage loci that we predicted were specific to the leaf, root, or flower tissue, selecting 10 loci for each tissue. Of the 30 tissue-specific candidate loci, 17 loci were functionally analyzed and previously reported to be expressed in the predicted tissue. Our RT-PCR results showed that all 30 tissue-specific candidate loci were expressed solely in the target tissues in cabbage. The RT-PCR results thus confirmed the reliability of our cabbage transcriptome assembly.

Our study provides valuable transcriptome sequence data for *B.* oleracea var. *capitata* L. and offers a resource for future studies of *B. oleracea* and closely related species. The assembled transcriptomic sequences and the annotation data will enhance the quality of the genome annotation and functional analysis of cabbage and serve as a material basis for future genomic researches of cabbage. Also the sequencing and annotation data from this study will be useful for developing molecular markers and identifying the extreme phenotypic differences and differential gene expression among members of the genus *Brassica*.

### Data deposition

The Illumina HiSeq2000 reads of *B.* oleracea var. *capitata* L. were submitted to NCBI Sequence Read Archive under the accession number of PRJNA227258.

## Supporting Information

Figure S1 Summary of Cabbage transcriptome assembly.
(TIF)

Table S1 Annotation of Cabbage transcriptome assembly.
(XLSX)

Table S2 GO terms of Cabbage transcriptome assembly.
(XLSX)

Table S3 KEGG annotation of Cabbage transcriptome assembly.
(XLSX)

Table S4 List of SNPs between two cabbage cultivars.
(XLSX)

Table S5 Tissue-specific locus candidates of Cabbage transcriptome assembly.
(XLSX)

Table S6 GO terms of Tissue-specific locus candidates.
(XLSX)

Table S7 Thirty tissue-specific locus candidates for RT-PCR.
(XLSX)

Table S8 Primer sets for RT-PCR.
(XLSX)
